# Supplementary material for: Attaching artificial Achilles and tibialis cranialis tendons to bone using suture anchors in a rabbit model: assessment of outcomes
Source: PeerJ. 2025 Jan 21;13:e18756. doi: 10.7717/peerj.18756 (PMC11758910; doi:10.7717/peerj.18756)
Supplement: Supplemental Information 2 [file peerj-13-18756-s002.docx]

**Supplemental Material**

Table 3. Previous studies involving artificial tendons and the method of attachment used.

| S/n | Year | Authors | Focus of the study | Suture anchor | Outcome |
| --- | --- | --- | --- | --- | --- |
| 1. | 2010 | (Melvin et al., 2010) | Replaced goat semitendinosus tendon with an artificial material (OrthoCoupler) | The method of attachment was not specifically stated. | Fatigue strength  of the OrthoCoupler was several times the contractile force of the semitendinosus muscle. |
| 2. | 2012 | (Melvin et al., 2012) | Replaced quadriceps tendon with the OrthoCoupler in a goat. | The OrthoCoupler was attached to a stainless steel bone plate on the tibia. | Mechanical testing of the myotendinous junction showed superior strength compared with the biological myotendon interface after 180 days. |
| 3. | 2024 | (Easton et al., 2024) | Replaced tibialis cranialis biological tendon with polyester silicone-coated artificial tendon in rabbits. | 2 mm × 6 mm bone suture anchor (Jorgenson Laboratories, Loveland, CO,  USA) , with a size 2 FiberWire suture (Arthrex Inc. Naples, FL). | The suture anchors held the artificial tendon securely to the bone in the rabbits and the artificial tendon restored normative biomechanical function in the rabbits. |
| 4. | 2024 | (Hsu et al., 2024) | Rotator cuff repair surgery on goat shoulders. | Custom-made MgF_2_-coated ZK60 suture anchor | Reestablished the connection between the detached infraspinatus tendon and the humeral head, with demonstrable osseointegration. |

Easton, Hatch, C., Stephens, K., Marler, D., Fidelis, O., Sun, X., Bowers, K. M., Billings, C., Greenacre, C. B., Anderson, D. E., & Crouch, D. L. (2024). Replacement of tibialis cranialis tendon with polyester, silicone-coated artificial tendon preserves biomechanical function in rabbits compared to tendon excision only. *Journal of Orthopaedic Surgery and Research*, *19*(1), 108. <https://doi.org/10.1186/s13018-024-04581-7>

Hsu, W.-C., Wu, G.-L., & Yeh, M.-L. (2024). Fixation technique of biodegradable magnesium alloy suture anchor in the rotator cuff repair of the shoulder in a goat model: a technical note. *BMC Musculoskeletal Disorders*, *25*(1), 246. <https://doi.org/10.1186/s12891-024-07300-9>

Melvin, A., Litsky, A., Mayerson, J., Witte, D., Melvin, D., & Juncosa-Melvin, N. (2010). An artificial tendon with durable muscle interface. *Journal of Orthopedic Research 28*(2), 218-224.

Melvin, A. J., Litsky, A. S., Mayerson, J. L., Stringer, K., & Juncosa-Melvin, N. (2012). Extended healing validation of an artificial tendon to connect the quadriceps muscle to the Tibia: 180-day study. *Journal of Orthopaedic Research*, *30*(7), 1112-1117.
